# Metagenome assembled genomes from hot sugarcane mill mud

**DOI:** 10.1128/mra.00418-25

**Published:** 2025-07-31

**Authors:** Erik Lapidus, Minori Uchimiya, Anthony Hay

**Affiliations:** 1 Department of Microbiology, Cornell University 5922, Ithaca, New York, USA; 2 United States Department of Agriculture, Agricultural Research Service, Southern Regional Research Center 57578 , New Orleans, Louisiana, USA; University of Maryland School of Medicine, Baltimore, Maryland, USA

**Keywords:** biomass, sugarcane, biofertilizers, soil, waste management, pretreatment

## Abstract

The genomes of six bacteria from the phylum Bacillota were assembled from metagenomic DNA extracted from sugarcane mill mud obtained immediately after heat treatment. These metagenome-assembled genomes were 94.5%–99.8% complete, with <2% contamination, and ranged from 2.6 Mb to 6.5 Mb, encoded 2573–5724 predicted proteins, and had G + C contents from 37% to 46%.

## ANNOUNCEMENT

Sugarcane mill mud contains many essential plant nutrients (1). Due to its nutrient and microbial makeup, it is an attractive alternative to chemical fertilizers (2). We previously reported on the microbiomes of mill muds and metagenome-assembled genomes (MAGs) from aged mill mud (1, 3, 4). Here, we describe MAGs from mud collected within hours of production from two sugarcane mills in Louisiana (LA2 and LA3) (1). Fresh, wet mud from each mill was stored at 4℃ within hours of collection. Aliquots were air-dried, and then both the wet and dried muds from each location were stored at −20℃. Four separate libraries (wet and dry from both mills) were prepared with the Nextera XT Kit (Illumina, San Diego, CA, USA) using DNA extracted with the EZNA Soil DNA Kit (Omega Biotek, Norcross, GA, USA) and sequenced via Illumina NextSeq2K using a NextSeq P3 300 bp (2 × 150) kit to produce 68,083,156 pair-ended reads with 9,866,243,574 bp.

The reads were uploaded to Kbase (5) and analyzed following the workflow described by Chivian et al. (6) with default parameters unless specified otherwise. Sequencing adaptors were removed using Trimmomatic-v0.36 (7). Read quality was measured using FastQC-v0.12.1 (8). Merge Reads Libraries-v1.2.2 (5) combined wet and dried reads from each mill mud. Genome assembly was performed on combined and uncombined libraries using MEGAHIT-v1.2.9 (9), metaSPAdes-v3.15.3 (10, 11), and IDBA-UD-v1.1.3 (12). The quality of the assemblies was compared with QUAST-v4.4 (13, 14). MEGAHIT assemblies were determined to be the best and were binned using MetaBAT2-v1.7 (15, 16), MaxBin2-v2.2.4 (17–19), and CONCOCT-v1.1 (20, 21). Bins were aggregated with DAS Tool-v1.1.2 (22–25), and then CheckM-v1.0.18 (26) assessed heterogeneity (single copy genes with 2+ alleles detected) and filtered to >90% completeness and <5% contamination. GTDB-Tk-v2.3.2 (27–29) classified the remaining MAGs. Bins unidentified at the species level were extracted and annotated using the NCBI Prokaryotic Genome Annotation Pipeline (30–32).

MAGs were all bacteria representing phyla previously found in mill mud: Actinomycetota, Bacillota, and Pseudomonadota (1, 3, 4). Of these, six were unique at the species level and are included in Table 1. They were from thermotolerant spore-forming genera in the class Bacilli, including uncharacterized species of four genera: *Sporolactobacillus*, *Ectobacillus*, *Paenisporosarcina*, *Paenibacillus*, and one *Candidatus* genus: *Pristimantibacillus*, a novel genus recently described from a MAG obtained from DNA extracted from a lignocellulolytic enrichment culture (33).

**TABLE 1:** MAG taxonomic rank of closest relative, characteristics, and GenBank accession number

Accession number	GTDB-TK Taxonomy^*a*^	NCBI taxonomy	Genome coverage	Completeness (%)	Contamination (%)	Heterogeneity (2+)^*b*^	Gc (%)	Annotation size (bp)	No. of contigs	N50	No. of putative proteins	Source^*c*^
JBHGUY000000000	d Bacteria; p Bacillota; c Bacilli; o Bacillales_K; f Sporolactobacillaceae; g Sporolactobacillus; s	*Sporolactobacillus* sp.	21.7	98.45	1.16	2	44.57	3,825,415	49	180,915	3,721	LA3-D SRR33394486
JBHGUQ000000000	d Bacteria; p Bacillota; c Bacilli; o Bacillales; f Bacillaceae_G; g Ectobacillus; s	*Ectobacillus* sp.	16.8	98.85	1.75	7	42.85	3,439,109	150	180,915	3,467	LA3-D SRR33394486
JBHGUR000000000	d Bacteria; p Bacillota; c Bacilli; o Bacillales; f Bacillaceae_G; g Ectobacillus; s	*Ectobacillus* sp.	37.7	98.85	1.62	6	42.77	3,529,666	114	51,460	3,411	LA3-W SRR33394487
JBHGUT000000000	d Bacteria; p Bacillota; c Bacilli; o Bacillales; f Planococcaceae; g Paenisporosarcina; s	*Paenisporosarcina* sp.	27.9	94.7	0.88	2	37.01	2,601,154	46	134695	2,573	LA2-W SRR33394489
JBHGUU000000000	d Bacteria; p Bacillota; c Bacilli; o Paenibacillales; f Paenibacillaceae; g Paenibacillus; s	*Paenibacillus* sp.	23.4	99.85	0.2	2	46.2	6,548,575	24	626,618	5,724	LA2-C SRR33394489 SRR33394488
JBHGUX000000000	d Bacteria; p Bacillota; c Bacilli; o Paenibacillales; f Paenibacillaceae; g Pristimantibacillus; s	*Candidatus Pristimantibacillus* sp.	21.7	99.71	0.52	6	44	6,493,315	95	131,230	5,857	LA2-C SRR33394489 SRR33394488

^*a*^ p, phylum; c, class; o, order; f, family; g, genus; s, species.

^*b*^ number of genes with multiple alleles.

^*c*^ W, wet; D, dry; C, combined.

Three MAGs from the same mill but different read libraries (wet (W), dry (D), and combined (C)) were identified as *Ectobacillus* sp. The MAG from the combined library had greater heterogeneity than the MAGs from either separate library (C:18 > W:6 or D:7), suggesting that the wet and dry MAGs had distinct alleles and were two separate genomes; both are included in Table 1. There are currently only five validly named species of *Ectobacillus* (34–38); thus, these MAGs significantly contribute to our understanding of the diversity within this genus.

## Data Availability

This Whole-Genome Shotgun project has been deposited in GenBank under accession no. PRJNA1140588. Biosample and assembly accession numbers are listed in Table 1. The raw sequence files are available on the Sequence Read Archive using the following accession numbers: SRR33394486 (LA2-W) ,SRR33394487 (LA3-W), ,SRR33394488 (LA2-D), and SRR33394489 (LA3-D).
